# The Impact of Vitamin D on Skin Aging

**DOI:** 10.3390/ijms22169097

**Published:** 2021-08-23

**Authors:** Georgeta Bocheva, Radomir M. Slominski, Andrzej T. Slominski

**Affiliations:** 1 Department of Pharmacology and Toxicology, Medical University of Sofia, 1431 Sofia, Bulgaria; 2 Department of Dermatology, University of Alabama at Birmingham, Birmingham, AL 35294, USA; radomir.slominski@gmail.com; 3 Graduate Biomedical Sciences Program, University of Alabama at Birmingham, Birmingham, AL 35294, USA; 4 Comprehensive Cancer Center, Cancer Chemoprevention Program, University of Alabama at Birmingham, Birmingham, AL 35294, USA; 5 Veteran Administration Medical Center, Birmingham, AL 35294, USA

**Keywords:** skin aging, photoaging, skin immune responses, vitamin D, vitamin D metabolites, photoprotection

## Abstract

The active metabolites of vitamin D_3_ (D_3_) and lumisterol (L_3_) exert a variety of antiaging and photoprotective effects on the skin. These are achieved through immunomodulation and include anti-inflammatory actions, regulation of keratinocytes proliferation, and differentiation programs to build the epidermal barrier necessary for maintaining skin homeostasis. In addition, they induce antioxidative responses, inhibit DNA damage and induce DNA repair mechanisms to attenuate premature skin aging and cancerogenesis. The mechanism of action would involve interaction with multiple nuclear receptors including VDR, AhR, LXR, reverse agonism on RORα and -γ, and nongenomic actions through 1,25D_3_-MARRS receptor and interaction with the nongenomic binding site of the VDR. Therefore, active forms of vitamin D_3_ including its canonical (1,25(OH)_2_D_3_) and noncanonical (CYP11A1-intitated) D_3_ derivatives as well as L_3_ derivatives are promising agents for the prevention, attenuation, or treatment of premature skin aging. They could be administrated orally and/or topically. Other forms of parenteral application of vitamin D_3_ precursor should be considered to avoid its predominant metabolism to 25(OH)D_3_ that is not recognized by CYP11A1 enzyme. The efficacy of topically applied vitamin D_3_ and L_3_ derivatives needs further clinical evaluation in future trials.

## 1. Introduction

Skin, like any other organs, undergoes progressive decline in its physiological, morphological, and functional features during aging [1,2,3,4]. The phenomenon of aging is natural and genetically predisposed. The functions of the skin are crucial for the homeostasis and survival. As the largest organ in the human body, the skin, together with the hypodermis (subcutaneous fat), is both the source and the target for several hormones and neuromediators [5,6,7,8,9,10,11,12,13,14,15,16,17], making it an independent peripheral endocrine organ [5,18]. The skin has also the capacity of producing the prohormone vitamin D and transforming it to active metabolites [19,20,21,22,23,24,25], which can exert several different effects on the main skin cells (keratinocytes and fibroblasts) [20,25,26,27,28,29] and immune cells [4,28,30,31] via the activation of the nuclear vitamin D receptor (VDR) [29,32,33,34,35]. Vitamin D plays a pivotal role in skin homeostasis contributing to its barrier function [20,29,36,37,38]. Moreover, as an essential part of a functioning immune system, active forms of vitamin D modulate the cutaneous immunity [8,30,39].

The gold standard of analyzing a vitamin D status is by measuring its major circulating metabolite, 25-hydroxyvitammin D_3_ (25(OH)D_3_), via high-performance liquid chromatography (HPLC) or liquid chromatography tandem–mass spectrometry (LC-MS/MS) [40,41,42]. Recently, a novel sensitive and specific LC-MS/MS method of the simultaneous measurement of 13 circulating metabolites of vitamin D_3_ and D_2_ was presented [43].

Importantly, subclinical (30–50 nmol/L) and clinical vitamin D deficiency (<30 nmol/L) in the general population has become a global problem worldwide [44,45,46,47,48]. Several physiological factors may influence vitamin D status, such as age, body mass index (BMI), skin type, pregnancy, and exclusive breastfeeding [49,50,51,52,53]. The genetic polymorphisms of some genes involved in skin pigmentation (*TYR*, *TYRP1*, *EXOC2*, and *DCT*) are also associated with 25(OH)D_3_ serum concentration [54]. Many environmental factors contribute to vitamin D deficiency, such as the winter season, inadequate sun exposure, and high latitude location [55,56]. Sun avoidance and air pollution are the main factors leading to insufficient UVB exposure. Moreover, ozone and particulate matter (PM) can directly affect the cutaneous production of vitamin D [57]. Additionally, air pollutants, persistent organic pollutants, and heavy metals can behave like endocrine-disrupting chemicals (EDCs), which may cause vitamin D deficiency directly or indirectly. The latter would be secondary to a weight gain, the dysregulation of parathyroid hormone and calcium homeostasis, and a thyroid dysfunction [57,58]. Increasing evidence suggests that smoking can also decrease the serum levels of 25(OH)D_3_ [57].

With advancing age, the capacity of the skin to produce vitamin D_3_ decreases (irrespective of the season), and the degradation of its active forms increases [59,60]. It was found that the concentration of the precursor of vitamin D_3_ in the skin, 7-dehydrocholesterol (7-DHC), declines approximately by 50% from age 20 to age 80 years [59]. Several other factors contribute to the vitamin D deficiency state in accelerated age, including limited sun exposure, insufficient dietary intake of vitamin D, or diseases causing malabsorption. The vitamin D deficiency, which is common in advanced age, can decrease the important physiological functions of the skin such as protection from the environment and prevention of cancer development [25,39,61,62,63,64,65,66].

In this review, we aimed to discuss the significance of vitamin D in the skin aging process.

## 2. Skin Aging—Your Skin Can Reveal Stories

Skin aging is a complex process that is influenced by the total exposure of both intrinsic and extrinsic factors over the human lifespan (skin exposome), which is responsible for the progressive morphological and functional alterations of the aged skin [1,67,68,69]. The main internal factors affecting the chronological (physiological) skin aging include a hormonal decline and changes in gene expression with advancing age [1]. In addition, the cutaneous regenerative potential decreases with the age due to the excessive senescence of keratinocytes, fibroblasts, and melanocytes over time, which contributes to skin aging [70,71,72]. The aged skin is characterized by fine wrinkles and atrophy with reduced elasticity. This chronological aging affects all skin areas but shows phenotypic differences among different anatomical regions, and it varies significantly within different populations [67,73]. The single nucleotide polymorphisms (SNPs) of the *MC1R* gene (main regulator of melanin pigmentation [74]) are significantly linked to a perceived facial age, providing a new molecular base of youthful looks [75].

The most prominent external stressors affecting skin and causing its premature aging include ultraviolet (UV) radiation [76,77], ambient pollutants [78,79,80,81,82], and smoking [57,83]. The continuous exposure of the skin to these environmental insults stimulates the production of reactive oxygen species (ROS) and generate oxidative stress [84,85]. The environmental factors can also cause an impairment of the epidermal barrier function [69] and alterations in skin microflora [86,87], leading to significant morbidity [2,88].

Ultraviolet radiation (UVR) is the most harmful external factor contributing to the cutaneous photodamage on the sun-exposed areas. Photoaged skin is presented as a dry, deep-wrinkled skin with rough texture, dyschromia and senile lentigines, vascular complications, etc. [68,89]. UVR decreases the expression of filaggrin that contributes to epidermal hydration, so its downregulation from UVR could explain the skin xerosis in photoaging [90]. Both UVA (315–400 nm) and UVB (280–315 nm) have been shown to contribute to photoaging, either by imbalanced ROS production or by direct DNA damage [83]. However, UVA is considered to play a major role in the aging process. UVA represents more than 80% of total daily UV irradiation and can penetrate 5–10 times deeper into the reticular dermis, where it can damage the extracellular matrix (ECM) more significantly than UVB [91]. Moreover, UVA exposure increases the expression of matrix metalloproteinases (MMPs), especially the expression of the collagenolytic enzyme MMP-1 in dermal fibroblasts, which acts as an important regulator in photoaging [92,93]. Additionally, the chronic UVA irradiation inhibits hyaluronan synthesis, thus altering the composition of proteoglycans in the dermis [94]. A long-term exposure of UVA is related to photoaging and photocancer due to an overproduction of ROS and reactive nitrogen species (RNS), which can disrupt both the nuclear and mitochondrial DNA [95,96]. UVB can penetrate only through the epidermis but is biologically more active. It also induces the transformation of 7-DHC to vitamin D_3_ [97,98]. UVB absorbed by DNA and RNA induces a formation of cyclobutane pyrimidine dimers (CPDs) and other photoproducts [99], thus inducing various solar signature mutations in specific genes, including the tumor suppressor gene p53 [100,101]. UVR induces an accumulation of p53 protein in the nucleus that in turn activates the transcription of genes responsible for cell cycle arrest allowing DNA repair, as well as causing an induction of apoptosis of the cells with unrepaired DNA damage [102,103]. Specific p53 mutations can be found in high rates, not only in actinic keratosis (precancerous state) and squamous cell carcinomas (60–90%) with typical UV signature, but also in the normal appearance of UV-exposed skin (about 75%), compared with a much lower rate of such mutations in healthy sun-protected skin (5% of all cases) [104].

Chronic sunlight exposure, together with the persistence of cellular senescence, can drive an impaired regenerative capacity of the skin, chronic inflammation, and photoaging, which correlates with enhanced cancer risk [77,105,106,107]. Thereby, photoaging results in premature skin aging. Although some aging mechanisms share several similarities or overlapping, photoaged skin differs from physiologically aged skin in the ECM changes. Photoaged skin is characterized by degraded collagen and accumulated aberrant elastin fibers and glycosaminoglycans, whereas physiologically aged skin is presented by the atrophy of dermal structures [108].

The negative impact of ambient pollutants on human health and the human skin is of growing concern [109]. Ozone (O_3_) from the smog and PM, primarily contacting with the skin, is capable to stimulate ROS production and generate oxidative stress, leading to phenotypic features of extrinsic aging [69]. It was found that chronic exposure to PM leads to pigment spots and deep nasolabial folds [110,111]. Moreover, ultrafine particles (<0.1 μm) can penetrate tissues and localize in the mitochondria, causing an aberrant mitochondrial function because of the oxidative processes [112]. Additionally, the photo-pollution exposure may aggravate UVR-mediated skin aging [113].

UVR, predominantly UVA, by the excessive amount of ROS activates the mitogen-activated protein kinases (MAPKs) and transcription factors such as nuclear factor erythroid 2-like (Nrf2), c-Jun-N-terminal kinase (JNK), and nuclear factor-κβ (NF-κB), and increases the transcription of MMPs [114]. Activated MMPs, together with the decreased expression of MMP inhibitors (TIMP), cause a dysregulation of the ECM homeostasis and a progressive damage of collagen and elastin [115]. Additionally, UVR impairs the endogenous antioxidant enzymes, leading to an increased oxidative damage of collagen. The destruction of ECM integrity is visualized as wrinkle appearance in photo-damaged skin [93]. The activation of redox-sensitive transcription factors, the activator protein-1 (AP-1) and NF-κβ, involved in wrinkle formation and inflammation, plays crucial roles in skin aging [88]. Both factors, NF-κβ and AP-1, are elevated within hours after cutaneous exposure to low-dose UVB. The upregulation of AP-1 suppresses the transforming growth factor β (TGF-β) receptors, which further blocks the synthesis of procollagen [116,117]. Additionally, activated AP-1 stimulates the degradation of collagen by MMPs and triggers the main activator of the inflammatory response NF-κβ [118]. NF-κβ signaling is a well-known regulator of tissue homeostasis, and its central role in skin aging was recently underlined [119]. The ROS-induced activation of NF-κβ drives an increase of proinflammatory cytokines and MMPs and decreases TGF-β and type I collagen synthesis [119]. The proinflammatory cytokines (interleukin (IL)-1, IL-6, and tumor necrosis factor (TNF)-α) stimulate inflammatory responses and enhance the activation of NF-κβ [93]. It was found that NF-κβ expression could increase in mitochondrial DNA (mtDNA)-depleter mice and after restoring the mitochondrial function, the NF-κβ expression could be reduced. These data confirm that NF-κβ signaling is a key mechanism contributing to the skin and hair follicle pathologies [120]. Due to the longer wavelength, UVA reaches the dermal fibroblasts in vivo with the activation of the Nrf2-mediated expression of antioxidant genes. Unlike UVA, UVB does not activate Nrf2 in skin cells or even appears to have an inhibitory effect [121]. However, vitamin D_3_ derivatives, which are products of UVB action, do activate Nrf2 signaling [122]. The endogenous Nrf2 is essential for the protection of skin cells against oxidative insults and for regulating the redox balance during skin aging [123,124]. Many in vitro and in vivo studies confirmed the importance of the transcription factor Nrf2 and its downstream signaling in UV protection [125,126].

Indeed, the human skin aging is mainly driven by oxidative events. An extensive ROS production and insufficient scavenging activity or a mitochondrial dysfunction are crucial events in oxidative stress-induced skin aging. The high levels of ROS lead to oxidative damage of lipids, proteins, genomic, and mtDNA, and also can deplete and damage the antioxidant defense systems of the skin (both non-enzymatic and enzymatic one) [85,127].

Accumulating evidence support a strong link between the mitochondrial dysfunction and the aging process [126]. Many studies demonstrate a decrease in mtDNA content and mitochondrial number during aging. It is thought that mitochondrial dysfunction plays a role in accelerated cellular senescence, seen in advancing age [128,129,130]. Furthermore, mitochondria are believed to contribute to 90% of generated ROS in the cells [95]. mtDNA, as an important target for ROS, is highly vulnerable to the oxidative damage and possesses inefficient DNA repair mechanisms [96,131]. The functional decline of the mitochondria leads to vicious cycle effect contributing to further enhancement of ROS production [127,132].

## 3. Effects of Vitamin D_3_ on the Skin

### 3.1. Impact Paths on the Skin

Excessive exposure to solar UVR accelerates skin aging and could trigger cutaneous cancerogenesis [133]. However, UVR plays a beneficial role in the regulation of many skin functions [56,77,134]. The same UVB, responsible for the increase of non-melanoma skin cancer, is required for vitamin D_3_ production in the skin that supplies more than 90% of the vitamin D_3_ body’s requirement [44,55,135]. In the skin, vitamin D_3_ is essential for the formation of the epidermal barrier and hair follicles, and its deficiency has been linked to many proliferative and inflammatory cutaneous disorders [20,29,44,136].

**Figure 1:**
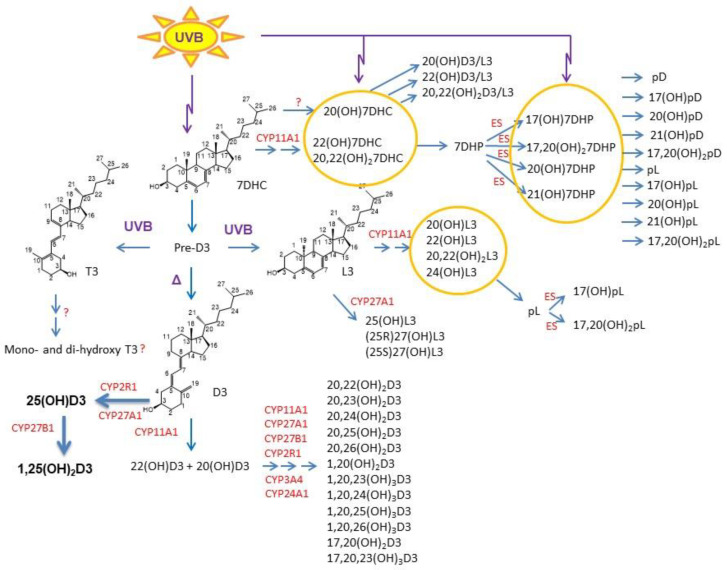
Noncanonical pathways of vitamin D_3_ and lumisterol (L_3_) activation (reprinted from [61] with the permission from Springer). D_3_, L_3_, and 7-DHC are substrates for CYP11A1 that produces the corresponding hydroxyderivatives. In the case of L_3_ and 7-DHC, the side chain can be cleaved by CYP11A1 to produce 7DHP or pL that can be further metabolized by steroidogenic enzymes (ES). In the skin, UVB acting on 5,7-dienes can lead to the production of D_3_, L_3_, and T_3_ derivatives with a full-length side chain and pD, pL, and pT derivatives with a shortened side chain. While the cut-off for UVC/UVB is 280 nm, we show the range of 290–315 nm because wavelengths below 290 nm are filtered by the ozone layer and no additional pre-D_3_ is produced above 315 nm [170]. 7DHC, 7-dehydrocholesterol; 7DHP, 7-dehydropregnenolone; pD, preganacalciferol; pL, preganalumisterol; D_3_, vitamin D_3_; L_3_, lumisterol; T_3_, tachysterol; OH, hydroxyl group; number before OH, carbon number with OH; number in subscripts after (OH), number of hydroxyl groups.

Upon the absorption of UVB, 7-DHC is transformed to vitamin D_3_ in the skin, a process accelerated by thermal energy. Prolonged UVB exposure can also generate tachysterol (T_3_) and lumisterol (L_3_) [24,97]. These reactions are non-enzymatic and dependent on the UVB dose and the temperature. Vitamin D_3_ can be activated through canonical and non-canonical pathways with similar activation of L_3_ to biologically active metabolites (Figure 1). In the classical pathway, vitamin D_3_ is hydroxylated to 25-hydroxyvitamin D_3_ (25(OH)D_3_) by CYP2R1 and/or CYP27A1 in the liver with further hydroxylation by CYP27B1 in the kidney, skin, and other tissues to its biologically active metabolite 1,25(OH)_2_D_3_ [20,21,137].

In the alternative (non-canonical) pathway, vitamin D_3_ can be activated by CYP11A1 with further modification by other cytochrome enzymes leading to production of large number of metabolites in humans [21,36,138,139,140,141,142] (Figure 1), some of which are non- or low-calcemic at high, therapeutic, doses [143,144,145,146]. The major CYP11A1-derived vitamin D_3_ products are 20(OH)D_3_ and 20,23(OH)_2_D_3_ [23,139,147,148]. In addition, 20(OH)D_3_ can be defined also as a natural product because its presence in the honey [149]. The L_3_ can also be metabolized to biologically active derivatives [150,151,152], which are not recognized by the 7-DHC reductase [153].

The main genomic effects and biological responses of vitamin D metabolites in the skin are mediated through their binding to the nuclear VDR [32,61,154,155,156]. Notably, VDR has been reported to regulate about 3% of mammalian genome due to its broad expression in all tissues [4,34,157,158]. The skin also expresses the VDR and serves not only as a source but also as a site for the action of vitamin D_3_ [28,39]. Additionally, the VDR activated by classical 1,25(OH)_2_D_3_ can induce rapid response signaling through a non-genomic, membrane-associated mechanism based on an alternative ligand-binding site [159] or through action on 1,25D_3_-MARRS receptor [156,160,161]. Similar non-genomic activities for CYP11A1-derived hydroxyderivatives are still not established. SNPs can affect VDR activity favoring a development of melanoma and non-melanoma skin tumors [162,163]. VDR functions as a tumor suppressor [164] and a decrease in its expression is associated with progression of cutaneous melanoma [165,166]. On the opposite, the nuclear VDR expression has been found significantly elevated (moderate to strong) in squamous cell carcinomas (SCCs) and basal cell carcinomas (BCCs) compared to in normal skin [167,168]. Thus, targeting VDR with vitamin D secosteroids (especially low calcemic ones) would be rational in skin cancer prevention, attenuation, or therapy [62,64,169].

CYP11A1-derived hydroxyderivatives can regulate some skin functions through other nuclear receptor such as retinoic acid-related orphan receptors (ROR) α and γ, which expressed in the skin [171]. The endogenously produced nonclassical vitamin D_3_ hydroxyderivatives, 20(OH)D_3_ and 20,23(OH)_2_D_3_ can act as weak RORα and RORγ inverse agonists [155,171,172]. Moreover, these hydroxyderivatives could exert anti-inflammatory effect and could inhibit tumor progression in the skin via RORγ-mediated mechanism [173].

Alternative, the classical 1,25(OH)_2_D_3_ and CYP11A1-initiated vitamin D_3_ derivatives can act as agonists on aryl hydrocarbon receptor (AhR) [174] and liver X receptors (LXR) [175]. Moreover, the activation of AhR is the top canonical pathway for 20,23(OH)_2_D_3_ [174]. This receptor regulates cellular proliferation, inflammation, and melanogenesis in the skin [176]. Although many different ligands can target AhR, some functional studies and molecular modeling can predict that secosteroidal signal transduction further leads to the downregulation of proinflammatory responses [177], detoxification, and antioxidative action [61,174].

**Figure 2:**
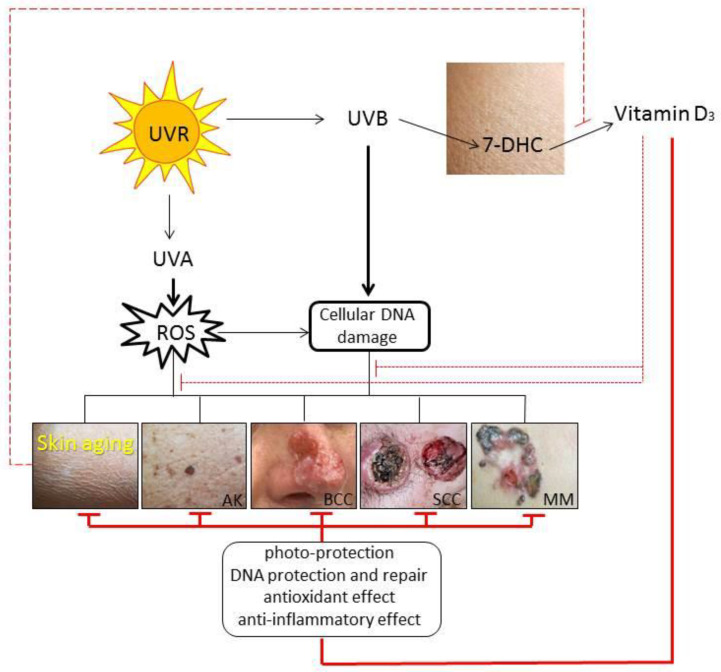
Photoprotective effects of vitamin D_3_ in premature skin aging and cutaneous cancerogenesis. Abbreviations: 7-DHC, 7-dehydrocholesterol; AK, actinic keratosis; BCC, basal cell carcinoma; SCC, squamous cell carcinoma; MM, malignant melanoma.

Summarizing, the biologically active classical and novel vitamin D_3_ metabolites exert different affinities to multiple receptors in the skin and through their modulation they can influence different cutaneous pathologies. In addition to act on the VDR, the active forms of vitamin D can act on alternative nuclear receptors including RORs, AhR, LXR, and 1,25D_3_-MARRS receptor. The active forms of vitamin D_3_ have various functions, which partially overlap in their anti-inflammatory, antimicrobial, antiproliferative, prodifferentiation, antifibrotic, and antioxidative effects on the skin [20,38,63,141,145,178]. Along with the best characterized 1,25(OH)_2_D_3_, CYP11A1-derived products of vitamin D_3_ and L_3_ exhibit photoprotective properties against UVR-induced skin damage (Figure 2) [37,61,179,180,181,182,183].

### 3.2. Effects on the Cutaneous Immune Function

Vitamin D_3_ and its analogues and precursors play an important complex role in the regulation of both the innate and adaptive immune systems, including in the skin [8,184,185]. There is a clear connection between vitamin D deficiency and incidences of infections and immune-mediated skin diseases [31,186,187,188]. The expression of the VDR has been found in almost all immune cells including T- and B-lymphocytes (Lym), macrophages, mast cells, natural killer (NK) cells, and regulatory T cells (Tregs), but it is differently regulated [189]. Monocytes, for example, lose VDR expression levels during differentiation towards macrophages and dendritic cells (DCs) [190,191]. Immunomodulatory actions of active vitamin D_3_ metabolites include the induction of Tregs [192] and Thelper-2 (Th2)-Lym, together with the downregulation of proinflammatory Th1/Th17/Th9-Lym [193]. 1,25(OH)_2_D_3_ can have both direct and indirect effects on T-Lym [194]. The indirect effect is based on DC-derived cytokines, which modulate Th-Lym response [30]. Directly, 1,25(OH)_2_D_3_ can suppress the immune cell production of inflammatory cytokines such as interferon gamma (IFN-γ), TNF-α, and IL-2 (Th1 cytokines), IL-17/21 (Th17 cytokines), and Th9 cytokines [193,195,196], while it enhances either the levels of anti-inflammatory IL-10 from Tregs [197] or Th2-derived IL-4 [198]. As a result, vitamin D shifts Th1 inflammatory response towards more tolerogenic Th2 response with an increase of CD4 + CD25 + Tregs reflected to a change in cytokine profile in the skin [199]. Additionally, 1,25(OH)_2_D_3_ influences the activated B-Lym by inducing apoptosis [200], suppressing immunoglobulin E (IgE)-dependent mast cell activation [201,202], and upregulating IL-10 production [203]. Enhanced IL-10 synthesis contributes to a suppressed mast cell-mediated inflammation and IgE-related allergic reactions [201]. 1,25(OH)_2_D_3_ and its analogues directly regulate antimicrobial peptide (AMP) gene expression in innate immune cells [204,205]. Moreover, it has been found that vitamin D is able to induce essential for antimicrobial defense, production of cathelicidin (LL37) [204] and to modulate the phagocytic activity of macrophages and NK cells [193]. Additionally, vitamin D exerts immunosuppressive effects through the modulation of epidermal Langerhans cells [206] and the proliferation of the Tregs number [184,185,197].

CYP11A1 is expressed also in immune cells [207], where vitamin D can be further metabolized to biologically active hydroxyderivatives [31,39]. Via the activation of VDR or through the inhibition of RORγ-mediated activation, 20(OH)D_3_ and 20,23(OH)_2_D_3_, like 1,25(OH)_2_D_3_, can attenuate Th17 differentiation, as well as the formation and activity of inflammatory cytokine IL-17 by immune cells [155,208,209]. Thus, Th17-related cutaneous inflammation could be successfully modulated via RORγ inverse agonists such as CYP11A1-derived D_3_-hydroxyderivatives, causing the regulation of the immune system and a resistance against autoimmunity [210,211]. The most recently inhibition of collagen-induced autoimmune arthritis by CYP11A1-derived 20(OH)D_3_ was reported [212].

### 3.3. Impact on Skin Aging

The normal vitamin D_3_ status is important for a general prevention of premature aging maintaining a healthful skin aging [213,214]. Vitamin D_3_ metabolites including its classical (1,25(OH)_2_D_3_) and novel (CYP11A1-intitated) D_3_ hydroxyderivatives exert many beneficial protective effects on the skin, which could influence the process of premature aging via many different mechanisms, leading to a delay or attenuation of both chronological skin aging and photoaging. Skin-resident cells (keratinocytes, fibroblasts, and sebocytes) are capable of locally activating vitamin D_3_ [23,36,215] and exhibiting a diverse biological effect such as photoprotection and immunosuppression, similar to the UVR-induced one [179,216].

The process of chronological aging is associated with immunological alteration and the imbalance between inflammatory and anti-inflammatory mechanisms, leading to a chronic low-grade inflammation, known as “inflammaging” state [217,218]. The “inflammaging” phenotype of the skin and hair follicles is a result of both chronic antigen stimulation and continued exposure to oxidative stress caused by ROS and RNS [219,220]. With advancing age, skin is affected by the profound remodeling of the immune system, leading to a decline in its adaptive capacity [221,222]. Th1- and Th17-related markers, together with the number of epidermal DCs are increased as a function of age [223,224,225]. DCs during aging appear to be functionally impaired, which contributes to an initiation of inflammatory and autoimmune skin disorders and a loss of their protective role against cutaneous infections. The active forms of vitamin D_3_ are able to decrease the proliferation and cytotoxicity of T-Lym, as well as to suppress the differentiation of B-Lym and the maturation of DCs [193]. Therefore, vitamin D_3_ hydroxyderivatives exert potent anti-inflammatory activities including the inhibition of TNF-α, INF-γ, and IL-1/6/9/17 production [4,38,185], suggesting their implication in the modulation of skin inflammation. Moreover, the noncalcemic and nontoxic 20(OH)D_3_ has shown a similar anti-inflammatory property in vivo to 1,25(OH)_2_D_3_ (hypercalcemic in high doses) through the suppression of the immune responses by T- and B-lym [155,212].

Active vitamin D_3_ metabolites can protect skin against the hazardous effects of skin aging-triggering agents, including UVR, pollution, and microbial infections [179,226,227,228,229,230]. It has been shown that the oral administration of high-dose vitamin D_3_ shortly after UVB exposure could reverse rapidly the photo-induced cutaneous damage by decreasing the inflammation and induction of the repair mechanisms of the epidermal barrier [38]. There is strong experimental evidence that active vitamin D_3_ and L_3_ hydroxyderivatives can induce, in a dose-dependent manner, antioxidative responses and reverse the UVB-mediated ROS production in keratinocytes by the activation of Nrf2 that works for cytoprotection and detoxification, thus attenuating photoaging [122]. Therefore, they serve as protective agents against UVB-induced oxidative stress in cells, pre-treated with each of these active metabolites for 24 h prior to UVB irradiation (50 mJ/cm^2^) [122]. These hydroxyderivatives stimulate the expression of antioxidant-response genes downstream of Nrf2 (*GR*, *HO-1*, *CAT*, *SOD-1*, and *SOD-2*) as well as the expression of HO-1, CAT, and MnSOD at the protein level [122]. The transcription factor Nrf2 plays an important role in the detection of excessive ROS and RNS and in the induction of mechanisms counteracting the oxidative damage and skin pigmentations produced by UVA [121,125,231].

Chronic UVR irradiation, mainly UVB [232] and UVA [233], induces DNA damage and the formation of CPDs that potentially lead to premature skin aging and carcinogenesis. CYP11A1-derived D_3_ and L_3_ hydroxyderivatives, along with 1,25(OH)_2_D_3_, demonstrate photoprotective and reparative properties by increasing the expression and phosphorylation of p53 with its translocation to the nucleus [61,229,234,235]. The P53 gene family, in particular its isoform p63, might be an important molecular target for vitamin D action in premature aging and cancer [236], which are promoted by similar mechanisms [237].

Moreover, 1,25(OH)_2_D_3_ and 1,25(OH)_2_L_3_ inhibit DNA damage and facilitate DNA repair by the reduction of CPDs [182,235,238,239] and RNS [178,234]. The photoprotection by 20(OH)D_3_ and 20,23(OH)_2_D_3_ is comparable to 1,25(OH)_2_D_3_ reduction of UVB-induced CPDs and DNA fragmentation in vivo [181,182] and in vitro [178]. In addition, both 20(OH)D_3_ and 20,23(OH)_2_D_3_ stimulate differentiation, inhibit proliferation and downregulate proinflammatory responses in keratinocytes via the decrease of NFκβ activity [240,241]. It was shown recently that not only the pretreatment, but also the post-treatment of keratinocytes with CYP11A1-derived D_3_ and L_3_ derivatives can reverse their UVB-induced damage [37,230].

Additionally, 1,25(OH)_2_D_3_ can induce rapid and dose-dependent reduction in skin cell apoptosis, and it can increase CPDs repair and decrease the oxidative DNA damage through non-genomic energy-conserving autophagy and mitophagy [227], thus contributing to the intrinsic skin photoprotection mechanism [242].

## 4. Conclusions and Future Perspectives

Vitamin D_3_ and its active metabolites exert a variety of antiaging and (photo) protective effects on the skin. These are achieved through immunomodulation that include anti-inflammatory actions and regulation of keratinocytes proliferation and differentiation program to build the epidermal barrier necessary to maintain skin homeostasis. In addition, they induce antioxidative responses, inhibit DNA damage and induce DNA repair mechanisms to attenuate premature skin aging and cancerogenesis. Similar actions can be assigned to lumisterol metabolites. Therefore, active forms of vitamin D_3_ including its canonical (1,25(OH)_2_D_3_) and noncanonical (CYP11A1-intitated) D_3_-hydroxyderivatives as well as L_3_-derivatives are promising agents for the prevention, attenuation, or treatment of premature skin aging, when applied topically. It is expected that they will attenuate photoaging and perhaps repair the existing damage induced by external stressors. The mechanism of action would involve interaction with nuclear receptors including VDR, AhR, LXR, reverse agonism on RORα and RORγ, and nongenomic actions through 1,25D_3_-MARRS receptor and interaction with the nongenomic binding site of the VDR. The regulatory mechanism affected by D_3_ and L_3_ derivatives would include the activation of Nrf2 and p53 and the downregulation of NFκβ signaling pathways or the regulation of mitochondrial functions. To prevent skin aging, vitamin D_3_ and lumisterol or their derivatives could be administrated orally and/or topically. Other forms of parenteral application of the vitamin D_3_ precursor should be considered to avoid channeling its metabolism to 25(OH)D_3_, which is not recognized by CYP11A1 enzyme [243]. The efficacy of topically applied vitamin D_3_ and L_3_ derivatives needs further clinical evaluation in future trials.

## Data Availability

Not applicable.
